# Retraction: LINC00152 down-regulated miR-193a-3p to enhance MCL1 expression and promote gastric cancer cells proliferation

**DOI:** 10.1042/BSR-2017-1607-T_RET

**Published:** 2023-02-27

**Keywords:** gastric cancer, LINC00152, MCL1, miR-193a-3p

This Retraction follows an Expression of Concern relating to this article previously published by Portland Press. This article is being retracted from *Bioscience Reports* at the request of the Editor-in-Chief and the Editorial Board following receipt of a notification from a reader, alerting the Editorial Board to duplications that appear in the article and between papers from unrelated authors. Specifically, the mouse tumour images from Figure 3A seem to appear as those in Figure 7A of Zhao et al. 2018 (doi: 10.1111/jcmm.13688) and in Figure 7A of Xue et al. 2018 (doi: 10.1111/jcmm.13604). The Editorial Office also identified a duplication between the Figure 5B BGC-823/NC and BGC-823/mimics+linc00152 images. The authors have been contacted with regards to the retraction and have not responded to the Journal's queries or the concerns raised. Given the extent of the issues raised, the Editorial Board stand by the decision to retract the article.

